# Monitoring Human Viral Pathogens Reveals Potential Hazard for Treated Wastewater Discharge or Reuse

**DOI:** 10.3389/fmicb.2022.836193

**Published:** 2022-04-08

**Authors:** Enric Cuevas-Ferrando, Alba Pérez-Cataluña, Irene Falcó, Walter Randazzo, Gloria Sánchez

**Affiliations:** Department of Preservation and Food Safety Technologies, Institute of Agrochemistry and Food Technology of the Spanish National Research Council (IATA-CSIC), Paterna, Spain

**Keywords:** enteric viruses, capsid integrity RT-qPCR, wastewater, crAssphage, fecal contamination indicator

## Abstract

Wastewater discharge to the environment or its reuse after sanitization poses a concern for public health given the risk of transmission of human viral diseases. However, estimating the viral infectivity along the wastewater cycle presents technical challenges and still remains underexplored. Recently, human-associated crAssphage has been investigated to serve as viral pathogen indicator to monitor fecal impacted water bodies, even though its assessment as biomarker for infectious enteric viruses has not been explored yet. To this end, the occurrence of potentially infectious norovirus genogroup I (GI), norovirus GII, hepatitis A virus (HAV), rotavirus A (RV), and human astrovirus (HAstV) along with crAssphage was investigated in influent and effluent water sampled in four wastewater treatment plants (WWTPs) over 1 year by a PMAxx-based capsid integrity RT-qPCR assay. Moreover, influent and effluent samples of a selected WWTP were additionally assayed by an *in situ* capture RT-qPCR assay (ISC-RT-qPCR) as estimate for viral infectivity in alternative to PMAxx-RT-qPCR. Overall, our results showed lower viral occurrence and concentration assessed by ISC-RT-qPCR than PMAxx-RT-qPCR. Occurrence of potentially infectious enteric virus was estimated by PMAxx-RT-qPCR as 88–94% in influent and 46–67% in effluent wastewaters with mean titers ranging from 4.77 to 5.89, and from 3.86 to 4.97 log_10_ GC/L, with the exception of HAV that was sporadically detected. All samples tested positive for crAssphage at concentration ranging from 7.41 to 9.99 log_10_ GC/L in influent and from 4.56 to 6.96 log_10_ GC/L in effluent wastewater, showing higher mean concentration than targeted enteric viruses. Data obtained by PMAxx-RT-qPCR showed that crAssphage strongly correlated with norovirus GII (ρ = 0.67, *p* < 0.05) and weakly with HAstV and RV (ρ = 0.25–0.30, *p* < 0.05) in influent samples. In effluent wastewater, weak (ρ = 0.27–0.38, *p* < 0.05) to moderate (ρ = 0.47–0.48, *p* < 0.05) correlations between crAssphage and targeted viruses were observed. Overall, these results corroborate crAssphage as an indicator for fecal contamination in wastewater but a poor marker for either viral occurrence and viral integrity/infectivity. Despite the viral load reductions detected in effluent compared to influent wastewaters, the estimates of viral infectivity based on viability molecular methods might pose a concern for (re)-using of treated water.

## Introduction

The microbiological analysis of raw and treated wastewater has become a hot topic in recent years due to the emerging concerns on the disease/pathogen epidemiological tracking (known as wastewater-based epidemiology, WBE) and on the safety of wastewater discarding and reusing. Monitoring of wastewater has already been implemented with success for a long time on the tracking of chemical pollutants, drug spread within communities, and antibiotic resistance genes (ARGs) (Mercan et al., 2019; de Oliveira et al., 2020). Over the last years, molecular analysis detection of viruses in wastewater samples has allowed disease surveillance as for poliovirus during the global eradication program (Asghar et al., 2014), re-emerging zoonotic pathogens such as hepatitis E virus (Miura et al., 2016; Cuevas-Ferrando et al., 2020), human enteric viruses (Hellmér et al., 2014; Prevost et al., 2015; Santiso-Bellón et al., 2020), and very recently severe acute respiratory syndrome coronavirus 2 (SARS-CoV-2) (Bivins et al., 2020b; Randazzo et al., 2020).

Human enteric viruses are the causative agents of viral gastroenteritis, hepatitis, and other diseases which predominantly transmit through the fecal–oral route (Oude Munnink and van der Hoek, 2016). Viral spread is mainly associated to person-to-person contact and ingestion of contaminated food and waters since enteric viruses are shed at huge concentrations of up to 10^13^ particles per gram by both symptomatic and asymptomatic individuals (Carter, 2005; Bosch et al., 2008; Okoh et al., 2010). Group A rotavirus (RV), norovirus genogroups I (GI) and II (GII), hepatitis A virus (HAV), and human astrovirus (HAstV) are the main causative agents of water−associated viral gastroenteritis and hepatitis outbreaks worldwide (Bosch et al., 2008).

Human enteric viruses show higher resistance to decontamination treatments generally applied by wastewater treatment plants (WWTPs) such as chlorination and UV radiation (Gerba et al., 2018). Consequently, reclamation in WWTP does not usually achieve total removal of viral particles from sewage (Ramírez-Castillo et al., 2015) and they are commonly found in effluent water samples analyses (Sano et al., 2016; Farkas et al., 2018).

In the context of the exacerbated water shortage, the use of reclaimed water for irrigation, recreational, or industrial applications has become a strategy to tackle this critical problem (Barcelo and Petrovic, 2011). From a public health perspective, monitoring not only the occurrence but also the infectivity of viral human pathogens may permit to estimate the adequacy of current water reclamation systems. To approach this issue, targeting specific human viral pathogens or a properly selected indicator constitutes alternative strategies to pursue.

Traditionally, methods based on cell culture have been used in clinical virology to test for viral infectivity, which show considerable limitations when applied in environmental samples because of the co-contamination of multiple virus species, the absence of permissive cell lines for certain viruses, and the cytotoxic effect of wastewater in cell culture (Gerba et al., 2018; Randazzo et al., 2018a). In recent years, there has been an enormous progress in detecting enteric viruses in water samples by real-time polymerase chain reaction (qPCR) methods (Katayama et al., 2008; Simmons and Xagoraraki, 2011; Farkas et al., 2018). Even so, molecular assays do not discriminate between viruses with infective capacity, inactivated viruses, and free genome. To overcome this limitation, viability markers and binding assays have been coupled to qPCR detection to evaluate the integrity of the viral capsid as estimate for viral infectivity also in water samples (Parshionikar et al., 2010; Kim et al., 2011; Coudray-Meunier et al., 2013; Prevost et al., 2016; Randazzo et al., 2016, 2018b,c; López-Gálvez et al., 2018; Tian et al., 2018; Leifels et al., 2019, 2020; Shirasaki et al., 2020; Canh et al., 2021a). Of note, such approach has been very recently implemented for SARS-CoV-2 detection in wastewater (Canh et al., 2021b; Cuevas-Ferrando et al., 2021).

Capsid integrity is a strong indicator of virus infectivity, as virions with an accessible genome yield reduced qPCR signals, improving the molecular estimation of virions (Leifels et al., 2020). As an alternative, fecal indicator bacteria (FIB) have traditionally been used to estimate fecal contamination of environmental waters, even though surveillance data demonstrated FIB may not always correlate with human enteric viruses (Sano et al., 2016; Amarasiri et al., 2017; Sidhu et al., 2017; Gerba et al., 2018). Recently, viruses such as crAssphage (cross-assembly phage), tobacco mosaic virus (TMV), and pepper mild mottle virus (PMMoV) have been suggested as indicators for either human fecal contamination and viral human pathogen removal throughout wastewater reclamations in the WWTPs (Kitajima et al., 2014; García-Aljaro et al., 2017; Farkas et al., 2019, 2020; Symonds et al., 2019; Bivins et al., 2020a; Tandukar et al., 2020; Wu et al., 2020).

Thus, the primary goal of this study was defining the occurrence of infectious human enteric viruses in influent and effluent wastewater by rapid molecular methods and, secondly, testing the hypothesis of whether crAssphage would serve as indicator for viral infectivity.

To this end, we monitored, by PMAxx-RT-qPCR over a 1-year period, the occurrence of intact capsid potentially infectious RNA enteric viruses (i.e., norovirus GI and GII, HAV, RV, and HAstV) and crAssphage in influent and effluent water samples collected from four WWTPs in the Valencian Region (Spain). Moreover, we compared *in situ* capture RT-qPCR (ISC-RT-qPCR) and PMAxx-RT-qPCR assays as alternative estimates for viral infectivity using influent and effluent samples of a selected WWTP longitudinally over a year. Finally, this work contributes on the expansion of the actual data pool on spatiotemporal viral monitoring studies in raw and treated wastewater and increases the significance of qPCR results for public health, economic, and QMRA purposes.

## Materials and Methods

### Sampling Site and Sample Collection

**FIGURE 1:**
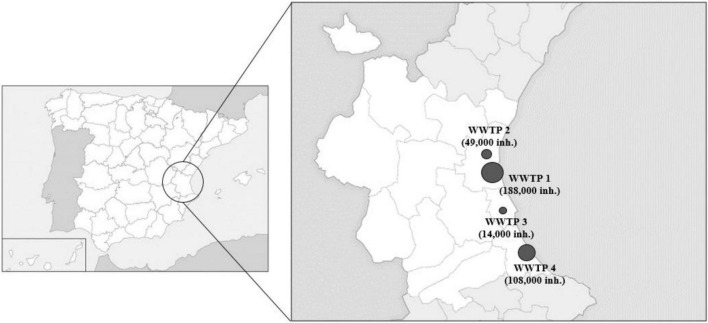
Geographical localization of wastewater treatment plants in Valencian region (Spain) and their population coverages included in the study. Diamonds on the figure are scaled according to population (inhabitants, inh).

Influent (*n* = 48) and effluent (*n* = 48) wastewater samples were collected regularly each month (from November 2018 to October 2019) from four WWTPs located in the Valencian region in Spain (Figure 1). Reclamation processes applied in each sampled wastewater treatment plant are described in Table 1. Samples were grabbed early in the morning (7–12 a.m.) by collecting 500–1,000 ml of water in sterile HDPE plastic containers (Labbox Labware, Spain). Collected samples were transferred on ice to the laboratory, kept refrigerated at 4°C and concentrated within 24 h as reported below.

**TABLE 1:** Reclamation processes of wastewater treatment plants.

Step	Treatment	WWTP 1	WWTP 2	WWTP 3	WWTP 4
	Coarse screening	X	X		X
	Fine screening	X		X	
Pretreatment	Sifting	X	X	X	X
	Flow homogenization tank	X			X
	Grit removal	X	X	X	X
	Grease removal	X	X	X	X
Primary	Physicochemical treatment	X			X
treatment	Decantation	X	X		X
	Activated sludge	X			X
	Extended aeration		X	X	
Secondary	Nitrogen removal	X	X	X	X
treatment	Phosphorus removal	X		X	
	Coagulation—Flocculation	X			
	Filtration	X			
Tertiary	UV	X		X	
treatment	Chlorination		X		X
					

### *Escherichia coli* Counts and Physicochemical Characterization of Influent and Effluent Wastewater Samples

Influent and effluent wastewater samples were characterized by determining significant physicochemical parameters. *Escherichia coli* was determined as the Most Probable Number (MPN)/100 ml according to EN ISO 9308-2 (2014). The total alkalinity (referred as TA) was determined by titration by measuring the UV-Vis absorbance following the methyl orange method and expressed in mg/L CaCO_3_. The chemical oxygen demand (COD, mg/L O_2_) was determined by measuring the UV-Vis absorbance on an AP3900 laboratory robot coupled with a DR3900 spectrophotometer (Hach) following the potassium dichromate method. Total suspended solids (TSS) were determined by filtration by using glass fiber filters and results expressed in mg/L. Turbidity (Nephelometric Turbidity Unit, NTU) were determined by TU5200 Laser Turbidimeter and the oxidation-reduction potential (ORP, expressed in mV) by HQ 40D digital multi meter (Hach, United Kingdom). Physicochemical analyses and *E. coli* counts were performed at GAMASER laboratories (Valencia, Spain).

### Virus Suspensions

Feces positive for norovirus GI, norovirus GII, and HAstV (courtesy of Dr. Buesa from Hospital Clínico Universitario, University of Valencia, Spain) were resuspended (10%, wt/vol) in phosphate-buffered saline (PBS) containing 2 M NaNO_3_ (Panreac, Spain), 1% beef extract (Conda, Spain), and 0.1% Triton X-100 (Thermo Fisher Scientific, Spain) (pH 7.2), vortexed and centrifuged at 1,000 × *g* for 5 min. The supernatants were extracted, the RNA stored at -80°C in aliquots to be used as positive amplification controls. The human RV strain Wa (ATCC VR-2018), the cytopathogenic HM-175 strain of HAV (ATCC VR-1402), and mengovirus vMC0 (CECT 100000) were propagated in MA-104, FRhK, and HeLa cell monolayers, respectively. Semipurified stocks were thereafter produced in the same cells by low-speed centrifugations of infected cell lysates (3,000 × *g* for 20 min). RNA extracted from infected cell lysates was used as positive amplification control and mengovirus (MgV) was used as process control as suggested in ISO 15216-2:2019 (microbiology of the food chain) for sample concentration validation (Randazzo et al., 2019).

### Wastewater Concentration

Influent and effluent water samples were artificially inoculated with approximately 7 log_10_ PCR units (PCRU)/L of MgV, as process control. Samples were concentrated through an aluminum hydroxide adsorption-precipitation method (AAVV, 2018; Randazzo et al., 2019). Briefly, 200 ml of sample was adjusted to pH 6.0 and Al(OH)_3_ precipitate formed by adding 1 part 0.9 N AlCl_3_ solution to 100 parts of sample. Then, pH was readjusted to 6.0 and sample mixed using an orbital shaker at 150 rpm for 15 min at room temperature. Next, viruses were collected by centrifugation at 1,700 × *g* for 20 min. The pellet was resuspended in 10 ml of 3% beef extract pH 7.4, and samples were shaken for 10 min at 150 rpm. Finally, the concentrate was recovered by centrifugation at 1,900 × *g* for 30 min and the pellet was resuspended in 1 ml of PBS and stored at -80°C.

### Viral Capsid Integrity Assays in Wastewater Samples

To assess the intact capsid condition of enteric viruses in influent and effluent wastewater, a main protocol based on capsid permeability to PMAxx viability dye (PMAxx-RT-qPCR) was used for all wastewater samples. Besides, an alternative method based on the specific binding ability to porcine gastric mucin (PGM) was run in parallel in samples from WWTP4 in order to evaluate its unreported capsid integrity discrimination efficiency on wastewater matrices.

For PMAxx-RT-qPCR, a previously optimized protocol was applied prior to nucleic acid extraction and RT-qPCR detection (Randazzo et al., 2016, 2018c; López-Gálvez et al., 2018). Briefly, the photoactivatable dye PMAxx™ (Biotium, United States) was added to 150 μl of each concentrated water sample at 50 μM along with 7.7 mmol/L Triton 100-X (Thermo Fisher Scientific, Spain) and incubated in the dark at room temperature for 10 min at 150 rpm. Later, samples placed in DNA LoBind 1.5 ml tubes (Eppendorf, Germany) were exposed to photoactivation using a Led-Active Blue system (GenIUL, Spain) for 15 min, and viral RNA was extracted and analyzed as described hereafter.

The *in situ* capture assay (ISC-RT-qPCR) was performed as previously reported (Wang and Tian, 2014; Falcó et al., 2019) in 24-well plates with some modifications. Briefly, each well was coated with 100 μl of PGM (100 μg/ml) in carbonate-bicarbonate buffer (pH 9.6) at 37°C for 1 h and then incubated overnight at 4°C. After being washed five times with 300 μl of PBS containing 0.05% Tween 20 and 0.3% BSA (PBSTB), wells were blocked with 300 μl of 3% BSA in PBS at 37°C for 2 h. Next, wells were washed five times with PBSTB, and 300 μl of concentrated water samples and controls were added to the 24-well plate and incubated at 37°C overnight. Untreated viral suspensions and those treated at 99°C for 5 min were used as positive and negative controls, respectively. Finally, after washing five times with PBSTB, 100 μl of lysis buffer from the NucleoSpin RNA virus kit (Macherey-Nagel GmbH and Co., Germany) was added to each well. Then, viral RNA was extracted and analyzed as described hereafter.

### RNA Extraction and Virus Quantification

Nucleic acids from each water sample were extracted following the NucleoSpin^®^ RNA virus kit (Macherey-Nagel GmbH and Co., Germany) manufacturer’s instructions with some modifications. In short, 150 μl of each concentrated sample was added with 25 μl Plant RNA Isolation Aid (Ambion, United Kingdom) and 600 μl of lysis buffer from the NucleoSpin^®^ RNA virus kit and subjected to pulse-vortexing. Then, the homogenate was centrifuged for 5 min at 10,000 × *g* for debris removal. The supernatant was subsequently processed according to the manufacturer’s instructions. Presence of norovirus GI and GII, HAV, HAstV, RV, and MgV was detected in 96-well plates using the RNA UltraSense One-Step kit (Invitrogen SA, United States), while crAssphage occurrence was performed using the qPCR Premix Ex Taq™ kit (Takara Bio Inc.) on the LightCycler^®^ 480 instrument (Roche Diagnostics, Switzerland). Moreover, undiluted and 10-fold diluted nucleic acid were tested in duplicate to check for inhibitors.

Different controls were used in all assays, including a concentration control to monitor the process efficiency of each sample (spiked MgV), a negative nucleic acid extraction control, and positive and negative RT-qPCR controls. Primers, probes, and RT-qPCR conditions used in this study are listed in Supplementary Table 1.

Standard curves were determined using the Public Health England (PHE) Reference Materials for Microbiology for norovirus GI (batch number 0122-17), norovirus GII (batch number 0247-17), and HAV (batch number 0261-2017) and reported as genomic copies (GC), while standard curves for RV, MgV, and HAstV were generated by amplifying 10-fold serial dilutions of viral suspensions in quintuplicates and calculating the number of PCR units (PCRU). Standard DNA material for crAssphage standard curve generation relied on a customized gBlock gene fragment (Integrated DNA Technologies, United States) containing target sequence for CPQ_064 crAssphage primers set (Stachler et al., 2017). All (RT)-qPCR determinations followed quality control and quality assurance criteria included in EMMI Guidelines (Borchardt et al., 2021).

### Statistical Analyses

Statistical data processing was performed using GraphPad Prism (GraphPad Software, La Jolla, CA, United States). The results were not normally distributed, so non-parametric Spearman’s rank correlation analyses were performed to evaluate the strength of relationship between viral titers alone, and in combination with physicochemical parameters. In all cases, values of *p* < 0.05 were deemed significant. Effects of wastewater treatment plant’s covered population, flow intake, and tertiary treatment (UV or chlorination) on crAssphage titers were analyzed by using the GraphPad Prism software. Correlation analyses among potentially infectious enteric viruses, crAssphage, *E. coli*, and physicochemical parameters were performed using viral loads, calculated as the product of viral titer per water flow for each WWTP. No log transformation was applied on data as Spearman’s rank correlation is invariant under monotone transformations like the logarithm.

## Results

### Occurrence of Intact Capsid Enteric Viruses and CrAssphage in Influent and Effluent Wastewater Over a 1-Year Period

**FIGURE 2:**
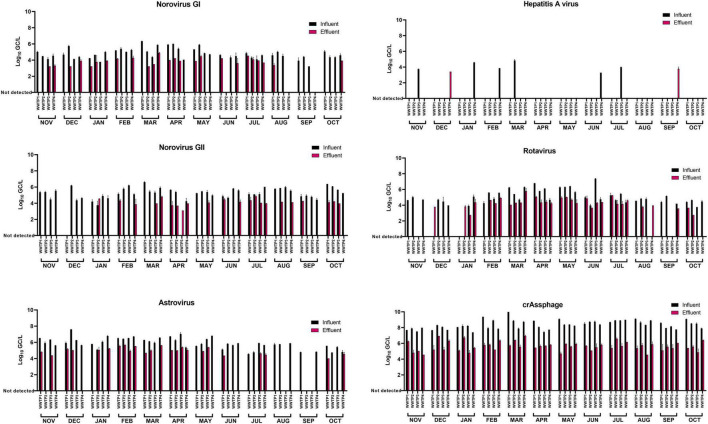
Occurrence of intact capsid enteric viruses and crAssphage in influent and effluent wastewater samples over a 1-year period. Capsid integrity was assessed by PMAxx-RT-qPCR. Colored bars represent mean Log_10_ GC/L values of two technical RT-qPCR replicates for each concentrated sample. Error bars indicate standard deviation.

Influent and effluent wastewater samples from four WWTPs located in the Valencian region (Spain) were processed by PMAxx-RT-qPCR over a 12-month period (2018–19) to determine the occurrence of potentially infectious norovirus GI, norovirus GII, HAV, RV, and HAstV, along with crAssphage (Figure 2).

It is worth to report that preliminary spiking experiments using murine norovirus (MNV, surrogate for human norovirus) and HAV assessed the effect of the wastewater concentration method on viral infectivity. According to the determination of the tissue culture infectious dose (TCID_50_/ml), no significant differences (*p* > 0.05) were observed among spiked and concentrated titers for both tested virus (data not shown).

The recoveries of MgV, spiked as viral process control, ranged between 1.18 and 37.80% (Supplementary Table 2); thus, results of targeted viruses were validated according to Haramoto et al. (2018) and the criteria included in the ISO 15216-1:2017 (recovery of control ≥1%). Viral titers of targeted viruses were not adjusted depending on the recovery of the concentration control (MgV) as back-calculation is not recommended (Haramoto et al., 2018). Norovirus GI, norovirus GII, and RV titers were 4.77 ± 0.65, 5.28 ± 0.63, and 5.08 ± 0.85 log_10_ GC/L in influent samples, and 3.86 ± 0.45, 4.13 ± 0.38, and 4.28 ± 0.64 log_10_ GC/L in effluent samples, respectively. HAstV showed the highest mean viral concentration among the five enteric RNA viruses in both influent (5.89 ± 0.68 log_10_ GC/L) and effluent (4.97 ± 0.43 log_10_ GC/L) samples. Moreover, HAstV was detected in 93.75% of influent water samples and in 50.0% of the effluent samples (Table 2). Overall, 93.8, 95.8, and 87.5% of influent samples (*n* = 48) and 52.1, 45.8, and 66.7% of effluent samples (*n* = 48) were positive for norovirus GI, norovirus GII, and RV, respectively. Finally, HAV was detected in 12 and 4.17% of the influent and effluent samples, respectively, and showed the lowest concentrations of 4.05 ± 0.56 log_10_ GC/L in influent samples and 3.60 ± 0.25 log_10_ GC/L in effluent samples. CrAssphage showed concentrations up to 3–4 log_10_ GC/L higher than targeted enteric RNA viruses ranging from 7.41 to 9.99 log_10_ GC/L in influent and from 4.56 to 6.96 log_10_ GC/L in effluent water samples, respectively. All samples tested positive for crAssphage and a mean decrease of 2.73 ± 0.68 log_10_ GC/L was observed in effluent compared to influent samples.

**TABLE 2:** Positive samples and percentiles of intact capsid enteric viruses assessed by PMAxx-RT-qPCR and *in situ* capture (ISC-)RT-qPCR assays in influent and effluent wastewater samples collected over a year from a selected wastewater treatment plant (WWTP4).

Virus	Wastewater sample	PMAxx-RT-qPCR	ISC-RT-qPCR
Norovirus GI	Influent (*n* = 12)	10 (83.3%)	7 (58.3%)
	Effluent (*n* = 12)	7 (58.3%)	3 (25%)
Norovirus GII	Influent (*n* = 12)	12 (100%)	9 (75%)
	Effluent (*n* = 12)	6 (50%)	1 (8.3%)
RV	Influent (*n* = 12)	11 (91.7%)	6 (50%)
	Effluent (*n* = 12)	9 (75%)	8 (66.7%)
HAstV	Influent (*n* = 12)	12 (100%)	12 (100%)
	Effluent (*n* = 12)	7 (58.3%)	6 (50%)

*RV, rotavirus; HAstV, human astroviruses.*

**FIGURE 3:**
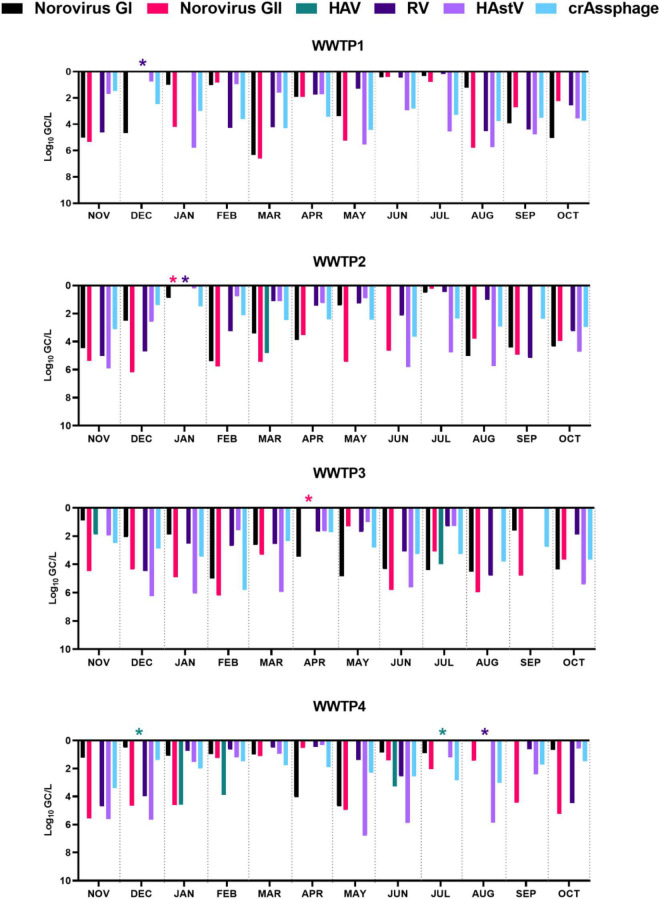
Enteric virus and crAssphage mean log removal (Log_10_ GC/L) for each wastewater treatment plant (WWTP) over a 1-year period. HAV, hepatitis A virus; RV, rotavirus; HAstV, human astroviruses; asterisks (*) indicate complete viral removal as viral load was higher in effluent than in influent wastewaters; missing bars are used for no detected virus in influent wastewaters.

Considering enteric virus, our data showed mean log removals of 2.58, 3.70, 2.39, and 3.08 for norovirus GI, norovirus GII, RV, and HAstV, respectively (Figure 3).

Viral removal separately calculated according to tertiary treatment, indicated log decreases of 3.65, 2.37, and 3.18 for norovirus GII, RV, and HAstV in UV-treated effluent wastewater (WWTP1 and WWTP3). Chlorination treatments in WWTP2 and WWTP4 determined log removals of 2.42, 2.97, and 3.75 for norovirus GII, RV, and HAstV. Viral removal differed between UV and chlorination showing 3.00 and 3.25 mean log reductions for UV, and 2.16 and 2.31 for chlorination for norovirus GI and crAssphage, respectively (Figure 3). None of the targeted viruses showed a sharp seasonal pattern (Figure 2). Extended data on viral quantification are presented in Supplementary Table 2.

### Comparing PMAxx-RT-qPCR and ISC-RT-qPCR Assays

In order to assess the efficiency of two alternative capsid integrity assays, PMAxx-RT-qPCR, and ISC-RT-qPCR were compared for detecting potential infectious norovirus GI, norovirus GII, HAstV, and RV in wastewater samples collected from a selected WWTP (*n* = 24). HAV was not tested by ISC-RT-qPCR because of its sporadic detection.

Overall, ISC-RT-qPCR provided lower estimates of viral occurrence than PMAxx-RT-qPCR for all tested viruses, except for HAstV, that showed 100% of positive samples in influent samples regardless of the capsid integrity assay applied (Table 2). Specifically, norovirus GI, norovirus GII, RV, and HAstV were detected by ISC-RT-qPCR in 58, 75, 50, and 100% of influent and in 25, 8, 67, and 50% of the effluent water samples. On the other hand, PMAxx-RT-qPCR estimated the occurrence of norovirus GI, norovirus GII, RV, and HAstV in 94, 96, 88, and 94% of influent and in 52, 46, 67, and 50% of effluent samples.

Regarding viral concentration, viral titers based on PMAxx-RT-qPCR assay resulted higher than those obtained by ISC-RT-qPCR in 93.75% determinations (Supplementary Figure 1).

### *Escherichia coli* Counts and Physicochemical Parameters

The *E. coli* counts and physicochemical parameters of influent and effluent wastewater samples are summarized in Supplementary Table 3. *E. coli* ranged from 3.96 to 8.19 log_10_ MPN/100 ml and from below the detection limit to 5.96 log_10_ MPN/100 ml in influent and effluent samples, respectively. Alkalimetric titration ranged from 58.30 to 744 mg/L CaCO_3_ and from 44.24 to 828 mg/L CaCO_3_ in influent and effluent samples, respectively. COD ranged from 28.7 to 5,768 and from 11.6 to 108 mg/l O_2_ in influent and effluent samples, respectively. Suspended solids ranged from 69.2 to 582.3 mg/l, and from 0.9 to 63.6 mg/l in influent and effluent samples, respectively. Turbidity values ranged from 0 to 247 units in influent and from 0 to 30.02 units in effluent samples. The redox potential ranged from 1.9 to 270.4 and from 1.2 to 224 mV in influent and effluent samples, respectively.

### CrAssphage as Fecal Viral Contamination Indicator of Potentially Infectious Enteric Viruses in Wastewater Samples

**FIGURE 4:**
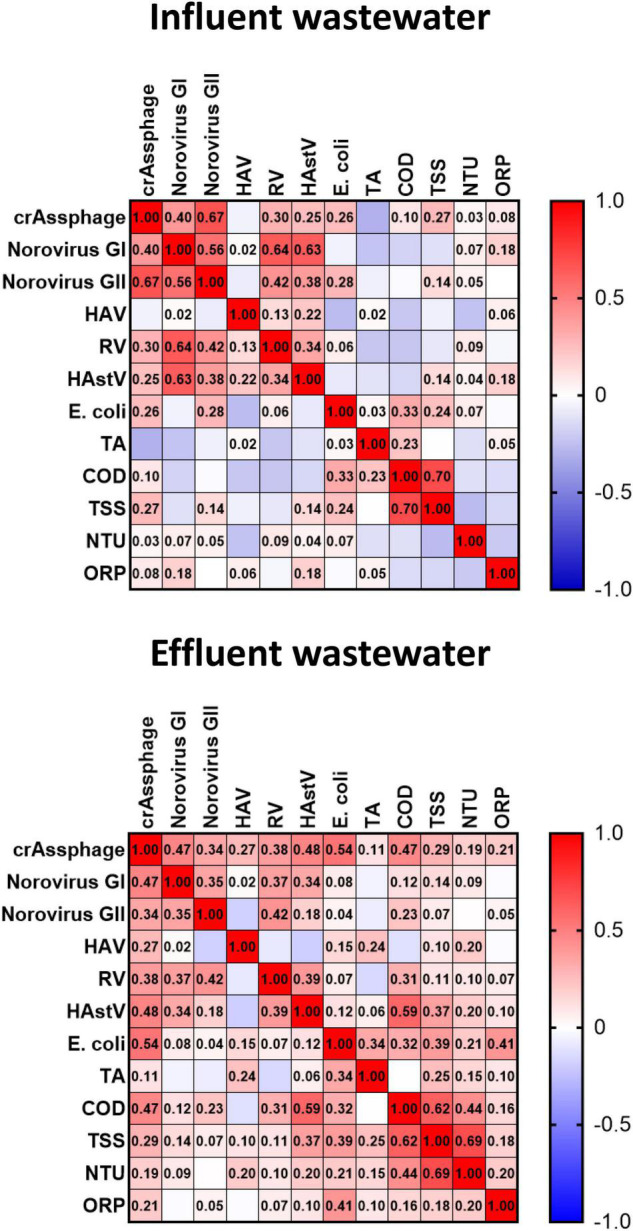
Spearman’s rho coefficients (ρ) of correlation analyses of intact capsid enteric viruses loads, crAssphage, and physicochemical parameters in influent and effluent wastewaters. TA, total alkalinity; COD, chemical oxygen demand; TSS, total suspended solids; NTU, turbidity; ORP, oxidation-reduction potential.

To further investigate the relationship among crAssphage, potentially infectious enteric virus, and physicochemical wastewater parameters, data sets were subjected to correlation analyses (Figure 4). Specifically, Spearman’s rank correlation rho coefficients (ρ) were calculated for intact capsid viral loads (viral titer × water flow) detected by PMAxx-RT-qPCR, *E. coli* counts, and physicochemical parameters in both influent (*n* = 48) and effluent (*n* = 48) wastewater samples (Figure 4). Resulting ρ coefficients are described through this work as follows: weak correlation (0.2–0.39), moderate correlation (0.4–0.59), strong correlation (0.6–0.79), and very strong correlation (0.8–1). In influent waters, crAssphage showed strong correlation with intact capsid norovirus GII (ρ = 0.67), moderate correlation with intact capsid norovirus GI (ρ = 0.40), and weak correlation with HAstV, RV, and *E. coli* (ρ = 0.25–0.30). Among enteric viruses, a moderate correlation resulted between norovirus GI and norovirus GII (ρ = 0.56). None to poor correlations resulted among enteric viruses and physicochemical parameters. When analyzing effluent wastewater samples, crAssphage showed moderate correlation with *E. coli* (ρ = 0.54) and intact capsid HAstV (ρ = 0.48) and norovirus GI (ρ = 0.47). Weak correlations resulted between crAssphage and RV (ρ = 0.38) and norovirus GII (ρ = 0.34). In contrast, *E. coli* displayed no correlation with any of the tested enteric viruses in effluent wastewater samples (ρ = 0.01–0.15).

### Effect of Wastewater Treatment Plant Characteristics on CrAssphage Load

The effect of key characteristics (population coverage, flow intake, and tertiary treatment) (Figure 1 and Table 1) on crAssphage load was independently evaluated for each WWTP.

CrAssphage load levels in influent (*p* = 0.039) and effluent (*p* = 0.007) wastewater statistically differed among WWTPs. Univariate results showed that variability of crAssphage load was statistically dependent on population coverage and on flow intake (*p* < 0.05) for influent wastewater samples. Interestingly, crAssphage concentration in effluent samples was found to significantly differ depending on the tertiary treatment (*p* = 0.016), suggesting that UV might be more efficient than chlorination for crAssphage removal. This was also observed for norovirus GI, but not for the remaining enteric virus (Figure 3).

## Discussion

The reuse of treated wastewater and its discharge into the environment poses a challenge for public health, as reclamation treatments needs to be adequate to provide water that is suitable for its intended purpose (e.g., irrigation, recreational, or drinking water). Governments and the scientific community agree on the need for monitoring the viral population in wastewater even though there is still much uncertainty on the analytical protocols to use as well as the load reduction needed for ensuring a minimal risk from exposure to reclaimed water (Gerba et al., 2018; Fenaux et al., 2019).

Capsid integrity is a strong indicator of virus infectivity and can be a worthy tool to adjust existent workflows and qPCR procedures to indicate the capability of viruses to infect humans, thus enhancing risk assessment inferred from monitoring programs (Leifels et al., 2020). However, the present investigation did not address the question on how to use data based on capsid integrity techniques as input for quantitative risk assessment; this query needs to be specifically explored in future work. A first step into this direction could be determining the relationships of viral infectious titers estimated by capsid integrity techniques and dose response resulting from clinical trials or known outbreaks.

### Presence of Potentially Infectious Enteric Viruses and Indicators in Wastewaters

This study provides additional insights on the quantitative occurrence of intact capsid enteric viruses in influent and effluent samples, and their correlation with crAssphage as a proposed viral water quality indicator.

We repetitively detected potentially infectious enteric viruses, including norovirus GI, norovirus GII, HAstV, and RV, in both influent and effluent in four different WWTPs over a year. This was further confirmed analyzing longitudinally upstream and downstream wastewater of a selected WWTP by two alternative capsid integrity assays, PMAxx-RT-qPCR and ISC-RT-qPCR, even though with different percentages (Table 2). Such release of human enteric viruses in effluent wastewater is not surprising as viral infectivity has been advised using different viability dye pretreatments (Gyawali and Hewitt, 2018; Randazzo et al., 2019; Canh et al., 2021a) and definitively demonstrated by cell culture (Simmons and Xagoraraki, 2011). However, comparing the viral titers determined by capsid integrity assays among WWTPs may not be conclusive due to different ratios of infectivity characterizing each population served by the sewerage system. This aspect could be additionally hindered for effluent wastewater samples exposed to different reclamation treatments (e.g., UV vs. chlorine) that distinctively affect viral morphology (e.g., nucleic acid vs. capsid), finally leading to diverse estimate of infectivity by capsid-integrity methods (Leifels et al., 2019).

Interestingly, a PMA-based capsid integrity assay was recently applied to assess the potential infectivity of novel HAV strains in treated wastewater in South Africa for which cell culture techniques may result to be not permissive (Rachida and Taylor, 2020).

Our results show that titers of viral particles with intact capsid in influent samples are comparable to those previously determined by RT-qPCR alone (Da Silva et al., 2007; Katayama et al., 2008; Kitajima et al., 2014; Montazeri et al., 2015; Haramoto et al., 2018), which suggests a high proportion of potentially infectious viruses, as expected.

However, capsid integrity RT-qPCR assays may not sharply discriminate infectious and inactivated viruses when subtle capsid alterations or genome damage occur because of the limited access to free RNA, the interaction with other compounds (e.g., organic acids), and the ineffective photoactivation (e.g., due to suspended solids, turbidity). These factors could differently affect capsid integrity RT-qPCR assays especially in complex matrices, such as wastewater, finally explaining the lower estimates for viral infectivity resulted from ISC-RT-qPCR compared to PMAxx-RT-qPCR. Thus, our findings further corroborate that PMAxx-RT-qPCR generally overestimate infectious viral particles (Leifels et al., 2015, 2020; López-Gálvez et al., 2018; Randazzo et al., 2018b, 2019). Nonetheless, capsid integrity RT-qPCRs better assess the potential risk of viral infection by providing more accurate information than conventional RT-qPCR alone that should be interpreted as a conservative approach.

### Reduction of Potentially Infectious Enteric Viruses and Indicators During Wastewater Treatments

We observed reductions of 2–3 log_10_ on average between upstream and downstream wastewater, which do not comply with the most recent European legislation. Specifically, a ≥ 6 log_10_ decrease of rotavirus, total coliphages, or at least one of them (F-specific or somatic coliphages) is indicated to validate monitoring programs of reclaimed water used for agricultural irrigation (Regulation (EU) 2020/741, 2020). However, specific guidelines should be defined globally as pointed out by the scientific community and water operators (Sano et al., 2016; Gerba et al., 2017).

In recent years, crAssphage has emerged as viral water quality indicator because of its specificity to human fecal pollution, its high concentrations in sewage, and its global presence (Farkas et al., 2019; Bivins et al., 2020a; Honap et al., 2020). Interestingly, KWR (Netherlands) included crAssphage to normalize SARS-CoV-2 titers in influent wastewater to monitor the COVID-19 pandemic (KWR, 2020), thus its potential as biomarker is not fully explored yet.

We detected crAssphage in all influent and effluent samples at mean concentrations of 8.37 ± 0.55 and 5.64 ± 0.59 log_10_ GC/L, respectively. These concentrations in influent wastewaters were roughly in line with the ones reported in the United Kingdom (Farkas et al., 2019), United States (Wu et al., 2020), and in a previous study conducted also in Spain (García-Aljaro et al., 2017). Slightly lower titers were reported in Thailand (Kongprajug et al., 2019) and in Italy (Crank et al., 2020). On the contrary, higher concentration of 10.98–12.03 log_10_ GC/L in influent and 7.45–8.62 log_10_ GC/L in effluent wastewaters were reported in Japan (Malla et al., 2019). These discrepancies might be due to the population served by WWTPs, the engineering characteristics of the sewer system (e.g., retention times, treatments, etc.), and the analytical method used for viral detection (wastewater concentration procedure, the genomic target, standards used to quantify viral concentrations), among other variables. Analyzing some of those variables, we observed statistically significant differences on crAssphage titers for served population, flow intake, and among WWTPs. This finding is in accordance to a previous report by Crank et al. (2020). Additionally, crAssphage concentrations in effluent wastewater were significantly lower when wastewater was exposed to UV than to chlorination. Thus, we further corroborate existing bibliography indicating the efficient viral disinfection applying UV light irradiation (Ali, 1997; Mezzanotte et al., 2007; Shah et al., 2011; Zyara et al., 2016). The increased mean removal in UV-treated wastewaters compared to chlorinated effluents can be extended to norovirus GI, but not for the other enteric viruses tested in this study (Figure 3).

### CrAssphage as Indicator for the Potential Infectivity of Enteric Viruses in Wastewater

The correlation between crAssphage and human viral pathogens has been reported in recent studies investigating wastewaters (Farkas et al., 2019; Malla et al., 2019; Crank et al., 2020; Tandukar et al., 2020), sludge (Wu et al., 2020), and other fecal polluted waters (Jennings et al., 2020). However, no information was available to date on whether crAssphage would serve as indicator for the potential infectivity of enteric viruses in wastewater. In influent wastewater, we found crAssphage strongly correlated to intact capsid norovirus GII and moderately to norovirus GI. In effluent wastewater, crAssphage moderately correlated with potentially infectious HAstV and norovirus GI.

Overall, the consistent detection of crAssphage in all influent and effluent samples corroborates the phage as an indicator for fecal contamination in wastewater. However, correlation readouts do not solidly support the use of crAssphage as indicator for the presence of potentially infectious enteric virus in wastewater, which was the primary hypothesis tested in this study. Thus, a strategy that targets each viral contaminant should be preferred to the sole detection of phages and this applies for both investigation and monitoring purposes.

The results of the present study also demonstrated that *E. coli*, adopted in the current regulation as fecal biomarker, and physicochemical parameters are not well suited as indicators for the viral contamination of wastewater, according to previous reports (Stachler et al., 2018; Ahmed et al., 2020).

### Limitation, Perspective, and Future Research

This study did not take into account environmental variables, such as rainfall and temperature, among others, that could have affected reported results.

Although analyzing the samples by RT-qPCR alone could have served as baseline to check the performance of PMAxx, previous studies already investigated the relationship of capsid integrity treatment on viral amplification signal reduction (Randazzo et al., 2016, 2019; Cuevas-Ferrando et al., 2020). Following a one-size capsid integrity treatment fits all approach and assuming it could lead to lower signal reduction (e.g., virus and matrix specificity: length and structure of genome targeted by the qPCR assays, the influence of co-concentrated inhibitory substances, etc.), we tested the hypothesis to adapt existent workflows for improving risk assessment.

Also, the comparison of molecular results with cell culture would have soundly confirmed our findings. However, viral cell culture of environmental samples presents technical challenges that are difficult to overcome (e.g., contamination, toxicity, sensitivity), especially in a longitudinal monitoring study such this one. A similar consideration can be done for crAssphage (Shkoporov et al., 2018).

Our findings based on capsid integrity assays could boost the development of advanced quantitative microbial risk assessment (QMRA) models for determining the risk of infection in case of treated wastewater reuse. This warrants further investigation and constitutes the gap to fill in the future in order to better quantify the human health risk, provide robust information for decision-making, and support water quality regulation.

In conclusion, this work provides insights on the quantitative occurrence of crAssphage and intact capsid enteric viruses in influent and effluent wastewater, while correlation outcomes indicated that crAssphage is a poor indicator for enteric virus infectivity in reclaimed wastewater.

## Data Availability Statement

The original raw dataset of the study are included as Supplementary Material in the article. Any further inquiries can be directed to the corresponding author.

## Author Contributions

GS and WR: conceptualization and funding acquisition. EC-F, AP-C, and IF: methodology. EC-F and WR: writing—original draft preparation. EC-F, AP-C, IF, WR, and GS: review and editing. All authors have read and agreed to the published version of the manuscript.

## Conflict of Interest

The authors declare that the research was conducted in the absence of any commercial or financial relationships that could be construed as a potential conflict of interest.

## Publisher’s Note

All claims expressed in this article are solely those of the authors and do not necessarily represent those of their affiliated organizations, or those of the publisher, the editors and the reviewers. Any product that may be evaluated in this article, or claim that may be made by its manufacturer, is not guaranteed or endorsed by the publisher.
