# Supplementary material for: Monitoring Human Viral Pathogens Reveals Potential Hazard for Treated Wastewater Discharge or Reuse
Source: Front Microbiol. 2022 Apr 8;13:836193. doi: 10.3389/fmicb.2022.836193 (PMC9026171; doi:10.3389/fmicb.2022.836193)
Supplement: Supplementary file 1 [file Data_Sheet_1.PDF]

Supplementary Material

Monitoring human viral pathogens reveals potential hazard for treated wastewater discharge or reuse

Enric Cuevas-Fernando[1], Alba Pérez-Cataluña[1], Irene Falcó[1], Walter Randazzo[1], Gloria Sánchez[1]*

[1]*Department of Preservation and Food Safety Technologies, Institute of Agrochemistry and Food Technology, IATA-CSIC, Av. Agustín Escardino 7, Paterna, 46980, Valencia, Spain.*

Table S1. Primers, probes and (RT)-qPCR conditions used in the study.

Virus	Primers and probe	Sequence	RT-qPCR conditions	Reference
NoV GI	QNIF4	CGC TGG ATG CGN TTC CAT	RT: 55 °C for 60 min, Preheating: 95 °C for 5 min PCR (45 cycles) 95 °C for 15 s, 60 °C for 60 s, 65 °C for 60 s.	(“ISO - ISO 15216-1:2017 - Microbiology of the food chain — Horizontal method for determination of hepatitis A virus and norovirus using real-time RT-PCR — Part 1: Method for quantification,” n.d.)
	NV1LCR	CCT TAG ACG CCA TCA TCA TTT AC		
	NVGG1p	[FAM]-TGG ACA GGA GAY CGC RAT CT-[BHQ]		
NoV GII	QNIF2	ATG TTC AGR TGG ATG AGR TTC TCW GA	RT: 55 °C for 60 min, Preheating: 95 °C for 5 min PCR (45 cycles) 95 °C for 15 s, 60 °C for 60 s, 65 °C for 60 s.	15216-1:2017. Microbiology of Food and Animal Feed — Horizontal Method for Determination of Hepatitis A Virus and Norovirus in Food Using Real-Time RT-PCR — Part 1: Method for Quantification,
	COG2R	TCG ACG CCA TCT TCA TTC ACA		
	QNIFs	[FAM]-AGC ACG TGG GAG GGC GAT CG-[BHQ]		
HAV	HAV68	TCA CCG CCG TTT GCC TAG	RT: 55 °C for 60 min, Preheating: 95 °C for 5 min PCR (45 cycles) 95 °C for 15 s, 60 °C for 60 s, 65 °C for 60 s.	15216-1:2017. Microbiology of Food and Animal Feed — Horizontal Method for Determination of Hepatitis A Virus and Norovirus in Food Using Real-Time RT-PCR — Part 1: Method for Quantification,
	HAV240	GGA GAG CCC TGG AAG AAA G		
	HAV150	[FAM]-CCT GAA CCT GCA GGA ATT AA-[MGBNFQ]		
RV	JVKF	CAG TGG TTG ATG CTC AAG ATG GA	RT: 50 °C for 30 min, Preheating: 95 °C for 15 min PCR (45 cycles) 94 °C for 10 s, 55 °C for 30 s, 72 °C for 20 s.	(Jothikumar et al., 2009)
	JVKR	TCA TTG TAA TCA TAT TGA ATA CCC A		
	JVKP	[FAM]-ACA ACT GCA GCT TCA AAA GAA GWG T-[BHQ]		
HAstV	AstVorf1b+	AAG CAG CTT CGT GAC TCT GG	RT: 55 °C for 60 min, Preheating: 95 °C for 5 min PCR (45 cycles) 95 °C for 15 s, 58 °C for 60 s, 65 °C for 60 s.	15216-1:2017. Microbiology of Food and Animal Feed — Horizontal Method for Determination of Hepatitis A Virus and Norovirus in Food Using Real-Time RT-PCR — Part 1: Method for Quantification,
	AstVorf1b-	AGC CAT CAC ACT TCT TTG GTC		
	AstVorf1bp	[FAM]-AGA GCA ACT CCA TCG CAT TT-[BHQ]		
MgV	Mengo 110	GCG GGT CCT GCC GAA AGT	RT: 55 °C for 60 min, Preheating: 95 °C for 5 min PCR (45 cycles) 95 °C for 15 s, 60 °C for 60 s, 65 °C for 60 s.	15216-1:2017. Microbiology of Food and Animal Feed — Horizontal Method for Determination of Hepatitis A Virus and Norovirus in Food Using Real-Time RT-PCR — Part 1: Method for Quantification,
	Mengo 209	GAA GTA ACA TAT AGA CAG ACG CAC AC		
	Mengo 147	[FAM]-ATC ACA TTA CTG GCC GAA GC-[MGBNFQ]		
crAssphage	064F1	TGT ATA GAT GCT GCT GCA ACT GTA CTC	Preheating: 95 °C for 5 min PCR (45 cycles) 95 °C for 5 s, 60 °C for 30 s	(Stachler et al., 2017)
	064R	CGT TGT TTT CAT CTT TAT CTT GTC CAT		
	064P1	[FAM]-CTG AAA TTG TTC ATA AGC AA-[MGBNFQ]		

Table S2. Intact capsid enteric viruses and crAssphage mean concentration values (log_{10} GC/L) and mengovirus recovery (%). *, indicate only one positive RT-qPCR replicate.

			Enteric viruses concentration (log_{10} GC/L)						
WWTP	Month	Type	Norovirus GI	Norovirus GII	HAV	RV	HAstV	crAssphage	MgV Recovery (%)
WWTP1	NOV	Influent	5.02 ± 0.08	5.35 ± 0.14	ND	4.62 ± 0.03	6.51 ± 0.01	7.7 ± 0.0	4.84
		Effluent	ND	ND	ND	ND	4.82 ± 0.01	6.24 ± 0.07	3.05
WWTP2		Influent	4.46 ± 0.03	5.38 ± 0.1	ND	5.02 ± 0.13	5.9 ± 0.17	7.9 ± 0.05	9.66
		Effluent	ND	ND	ND	ND	ND	4.81 ± 0.35	1.66
WWTP3		Influent	4.12 ± 0.22	4.47 ± 0.19	3.77 ± 0	ND	6.33 ± 0.02	7.5 ± 0.01	3.67
		Effluent	3.23*	ND	ND	ND	4.36 ± 0.01	5.03*	3.65
WWTP4		Influent	4.54 ± 0.22	5.55 ± 0.14	ND	4.69 ± 0.02	5.61 ± 0.02	7.97 ± 0.01	5.25
		Effluent	3.32 ± 0.13	ND	ND	ND	ND	4.56*	1.94
WWTP1	DEC	Influent	4.67 ± 0.2	ND	ND	ND	5.92 ± 0.12	7.66 ± 0.03	1.83
		Effluent	ND	ND	ND	3.79*	5.15 ± 0.11	5.2 ± 0.53	1.66
WWTP2		Influent	5.73 ± 0.11	6.19 ± 0.04	ND	4.71 ± 0.07	7.6*	8.32*	15.15
		Effluent	3.23*	ND	ND	ND	5.01 ± 0.04	6.92 ± 0.01	1.57
WWTP3		Influent	4.14*	4.35 ± 0.17	ND	4.46 ± 0.55	6.25 ± 0.02	8.08 ± 0.04	1.91
		Effluent	ND	ND	ND	ND	ND	5.21 ± 0.31	4.53
WWTP4		Influent	4.4 ± 0.06	4.64 ± 0.09	ND	3.98*	5.64 ± 0.03	7.71*	1.21
		Effluent	3.91 ± 0.16	ND	3.42 ± 0	ND	ND	6.33 ± 0.13	3.31
WWTP1	JAN	Influent	4.23 ± 0.01	4.21 ± 0.25	ND	ND	5.78 ± 0.02	8.06*	1.68
		Effluent	3.23	ND	ND	ND	ND	5.09 ± 0.07	10.43
WWTP2		Influent	4.65*	3.75 ± 0.5	ND	ND	5.09 ± 0.08	8.2 ± 0.17	1.40
		Effluent	3.78 ± 0.79	4.55 ± 0.03	ND	3.84 ± 0.16	5.05 ± 0.35	6.73 ± 0.17	1.49
WWTP3		Influent	3.78*	4.89 ± 0.17	ND	3.91 ± 0.13	6.06 ± 0.11	8.22 ± 0.03	1.32
		Effluent	ND	ND	ND	2.75*	ND	4.79 ± 0.33	5.31
WWTP4		Influent	5 ± 0.08	4.61 ± 0.32	4.58 ± 0.04	5.09 ± 0.22	6.77 ± 0.07	7.41*	13.20
		Effluent	3.91 ± 0.02	ND	ND	4.36 ± 0.37	5.23 ± 0.01	5.42 ± 0.07	5.85
WWTP1	FEB	Influent	5.19 ± 0.02	5.15 ± 0.14	ND	4.26 ± 0.21	6.49 ± 0.05	9.36*	2.10
		Effluent	4.17 ± 0.04	4.32 ± 0.16	ND	ND	5.54 ± 0.11	5.79 ± 0.23	12.44
WWTP2		Influent	5.39 ± 0.16	5.78 ± 0.11	ND	5.58 ± 0.04	6.43 ± 0.02	7.93 ± 0.11	10.88
		Effluent	ND	ND	ND	4.65*	5.66 ± 0.04	5.83 ± 0.09	2.15
WWTP3		Influent	5.01 ± 0.03	6.2 ± 0.06	ND	4.81 ± 0.18	6.51 ± 0.01	8.91 ± 0.05	12.00
		Effluent	ND	ND	ND	4.24*	4.92 ± 0.13	5.2*	15.02
WWTP4		Influent	5.25 ± 0.07	5.11 ± 0.07	3.87 ± 0	5.56 ± 0.01	6.71 ± 0.04	7.84 ± 0.03	10.88
		Effluent	4.29 ± 0.21	3.86 ± 0.65	ND	4.91 ± 0.1	5.51 ± 0.02	6.37 ± 0.11	9.54
WWTP1	MAR	Influent	6.34 ± 0.01	6.61 ± 0.07	ND	6.24 ± 0.04	6.27 ± 0.03	9.99 ± 0.03	3.58
		Effluent	ND	ND	ND	4.04*	4.67 ± 0.01	5.72 ± 0.08	4.33

WWTP2		Influent	5.04 ± 0.06	5.44 ± 0.13	4.83 ± 0.13	5.41*	6.11 ± 0.02	8.86 ± 0.07	1.53
		Effluent	3.23*	ND	ND	4.28 ± 0.03	4.99 ± 0.08	6.41 ± 0.03	1.45
WWTP3		Influent	4.39 ± 0.06	5.31 ± 0.2	ND	4.71 ± 0.09	5.94 ± 0.11	7.88 ± 0.07	5.05
		Effluent	3.5*	ND	ND	4.33*	ND	5.55 ± 0.26	19.16
WWTP4		Influent	5.87 ± 0.03	5.91 ± 0.12	ND	6.3 ± 0.03	6.57 ± 0.04	8.72 ± 0.07	10.39
		Effluent	4.89 ± 0.09	4.8 ± 0.09	ND	5.79 ± 0.35	5.62 ± 0.01	6.96 ± 0.07	14.73
WWTP1	APR	Influent	5.9 ± 0.05	5.65 ± 0.03	ND	6.8 ± 0.01	6.72 ± 0.02	8.85 ± 0.03	10.13
		Effluent	3.98 ± 0.03	3.74 ± 0.48	ND	5.06 ± 0.09	5 ± 0.03	5.43 ± 0.01	10.12
WWTP2		Influent	6 ± 0.01	5.39 ± 0.1	ND	5.79 ± 0.01	6.25 ± 0.08	8.06 ± 0.04	11.61
		Effluent	4.23*	3.69*	ND	4.34 ± 0.34	4.98 ± 0.01	5.65 ± 0.15	7.70
WWTP3		Influent	5.4 ± 0.1	ND	ND	6.12 ± 0.01	7.03 ± 0.19	7.42 ± 0.11	3.64
		Effluent	3.91*	3.1*	ND	4.43 ± 0.2	5.38 ± 0.06	5.7 ± 0.02	4.92
WWTP4		Influent	4.05*	4.24 ± 0.38	ND	4.68 ± 0.19	5.35 ± 0.07	7.72 ± 0.05	3.65
		Effluent	ND	3.98*	ND	4.23 ± 0.16	5.03 ± 0.19	5.82 ± 0.03	2.89
WWTP1	MAY	Influent	5.33*	5.24 ± 0.02	ND	6.26 ± 0.07	5.54 ± 0.04	9.09 ± 0.03	9.15
		Effluent	3.88 *	ND	ND	4.96 ± 0.15	ND	4.7 ± 0.19	5.21
WWTP2		Influent	5.89 ± 0.06	5.45 ± 0.02	ND	6.31*	5.79 ± 0.1	8.36 ± 0.05	12.61
		Effluent	4.47 ± 0.12	ND	ND	5.02 ± 0.03	4.89 ± 0.02	5.94 ± 0.02	7.61
WWTP3		Influent	4.85 ± 0.03	5.39 ± 0.2	ND	6.39 ± 0.04	6.39 ± 0.01	8.39 ± 0.01	14.00
		Effluent	ND	4.08 ± 0.24	ND	4.69 ± 0.14	5.38 ± 0.01	5.59 ± 0.07	10.04
WWTP4		Influent	4.69 ± 0.04	4.96 ± 0.11	ND	5.64 ± 0.09	6.78 ± 0.04	8.23 ± 0.01	10.98
		Effluent	ND	ND	ND	4.26 ± 0.45	ND	5.93 ± 0.11	4.00
WWTP1	JUNE	Influent	4.32 ± 0.07	4.85 ± 0.15	ND	4.99 ± 0.14	5.12 ± 0.07	8.49 ± 0.16	12.76
		Effluent	4.2 ± 0.03	4.46 ± 0.14	ND	4.85 ± 0.32	4.36*	5.69 ± 0.03	19.33
WWTP2		Influent	ND	4.66 ± 0.11	ND	3.96 ± 0.15	5.82 ± 0.02	8.72 ± 0.07	2.41
		Effluent	ND	ND	ND	3.66*	ND	5.09*	3.70
WWTP3		Influent	4.32 ± 0.21	5.8 ± 0.04	ND	7.37 ± 0.04	5.63 ± 0.04	8.76 ± 0.09	17.65
		Effluent	ND	ND	ND	4.28 ± 0.08	ND	5.5 ± 0.44	34.80
WWTP4		Influent	4.49 ± 0.39	5.58 ± 0.11	3.28*	4.75 ± 0.22	5.89*	8.37 ± 0.04	3.34
		Effluent	3.63 ± 0.57	4.18 ± 0.23	ND	4.38*	ND	5.82 ± 0.11	5.61
WWTP1	JULY	Influent	4.86 ± 0.14	5.12 ± 0.18	ND	5.25 ± 0.27	4.55 ± 0.06	8.68 ± 0.04	6.03
		Effluent	4.52 ± 0.01	4.35 ± 0.38	ND	5.24 ± 0.01	0 ± 0	5.41 ± 0.35	25.66
WWTP2		Influent	4.3 ± 0.13	5.07 ± 0.04	ND	4.63 ± 0.08	4.77 ± 0.1	8.9 ± 0.04	1.29
		Effluent	4.08 ± 0.29	4.85 ± 0.16	ND	4.17 ± 0.45	ND	6.58 ± 0.11	28.65
WWTP3		Influent	4.1 ± 0.42	5.11 ± 0.2	3.99 ± 0.03	5.44 ± 0.06	5.9*	8.9 ± 0.01	7.86
		Effluent	3.95*	4.04*	ND	4.14 ± 0.33	4.63 ± 0.11	5.65 ± 0.31	8.96
WWTP4		Influent	4.6 ± 0.07	6.01*	ND	4.36 ± 0.3	5.67*	8.99*	4.78
		Effluent	3.7*	3.97*	ND	4.6 ± 0.08	4.47 ± 0.15	6.15 ± 0.05	11.17
WWTP1	AUG	Influent	4.59 ± 0.24	5.78 ± 0.02	ND	4.54 ± 0.03	5.74 ± 0.15	9.1 ± 0.04	6.00
		Effluent	3.36 ± 0.19	ND	ND	ND	ND	5.37 ± 0.3	2.32
WWTP2		Influent	5.02 ± 0.08	5.88*	ND	4.82 ± 0.08	5.76 ± 0.02	8.66 ± 0.09	5.76

		**Effluent**	ND	4.18*	ND	3.8 ± 0.09	ND	5.76 ± 0.23	8.81
**WWTP3**		**Influent**	4.52 ± 0.23	5.98 ± 0.13	ND	4.8 ± 0.15	ND	8.33 ± 0.02	9.05
		**Effluent**	ND	ND	ND	ND	ND	4.56*	7.43
**WWTP4**		**Influent**	ND	5.55 ± 0.1	ND	ND	5.87 ± 0.01	8.89 ± 0.03	4.14
		**Effluent**	ND	4.11*	ND	3.96*	4.64 ± 0.08	5.88 ± 0.29	3.42
**WWTP1**	**SEPT**	**Influent**	3.93 ± 0.35	4.85 ± 0.25	ND	4.4 ± 0.12	4.78 ± 0.03	8.59 ± 0.05	2.04
		**Effluent**	ND	4.27*	ND	ND	ND	5.1 ± 0.76	5.22
**WWTP2**		**Influent**	4.41 ± 0.07	4.93 ± 0.12	ND	5.16 ± 0.03	ND	7.94 ± 0.01	3.67
		**Effluent**	ND	ND	ND	ND	ND	5.57 ± 0.24	3.66
**WWTP3**		**Influent**	3.23*	4.78 ± 0.11	ND	ND	ND	8.13 ± 0.04	6.82
		**Effluent**	ND	ND	ND	ND	ND	5.38 ± 0.47	16.02
**WWTP4**		**Influent**	ND	4.43 ± 0.2	ND	4.19*	4.83 ± 0.02	7.75*	1.89
		**Effluent**	ND	ND	3.77 ± 0.23	3.58*	ND	6.05*	2.51
**WWTP1**	**OCT**	**Influent**	5.05 ± 0.15	6.36 ± 0.01	ND	4.39 ± 0.09	5.56*	9.09 ± 0.01	4.88
		**Effluent**	ND	4.11 ± 0.23	ND	3.67*	4.01*	5.39 ± 0.02	10.24
**WWTP2**		**Influent**	4.35 ± 0.18	6.08 ± 0.01	ND	4.64 ± 0.04	4.71 ± 0.04	8.51 ± 0.03	2.39
		**Effluent**	ND	4.23*	ND	2.75*	ND	5.57 ± 0.15	1.97
**WWTP3**		**Influent**	4.36 ± 0.07	5.65*	ND	3.76*	5.41 ± 0.1	8.5 ± 0.11	4.43
		**Effluent**	ND	3.98*	ND	ND	ND	4.87 ± 0.43	4.69
**WWTP4**		**Influent**	4.6 ± 0.19	5.23 ± 0.02	ND	4.47 ± 0.12	4.81 ± 0.2	7.89 ± 0.05	2.76
		**Effluent**	3.94*	ND	ND	ND	4.54 ± 0.24	6.39 ± 0.05	2.26

Table S3. Physicochemical characterization of influent and effluent wastewater samples. Abbreviations: MPN, most probable number; COD, chemical oxygen demand; NTU, Nephelometric Turbidity Unit; NA, data no available.

WWTP	Month	Type	*E. coli* (MPN/100mL)	ALKALIMETRIC TITRATION (mg/L $CaCO_3$)	COD (mg/L O_2)	SUSPENDED SOLIDS (mg/L)	TURBIDITY (NTU)	REDOX POTENTIAL (mV)
WWTP1	NOV	Influent	111990	NA	NA	NA	NA	NA
		Effluent	77010	503	30	3	2	162.0
WWTP2		Influent	1935000	NA	NA	NA	NA	NA
		Effluent	22820	278	31	7	3	224.0
WWTP3		Influent	5200000	644	520	260	0	270.40
		Effluent	5	469	18	3	2	23.1
WWTP4		Influent	12997000	614	76	202	37	184.00
		Effluent	155310	589	23	7	1	29.4
WWTP1	DEC	Influent	4611000	579	457	162	44	45.40
		Effluent	3	490	35	4	2	6.2
WWTP2		Influent	129970000	745	682	357	0	54.6
		Effluent	64880	485	59	30	12	49.2
WWTP3		Influent	15531000	544	648	218	88	1.90
		Effluent	0	443	17	5	2	2.2
WWTP4		Influent	6131000	515	41	108	53	3.6
		Effluent	198630	506	22	9	4	3.3
WWTP1	JAN	Influent	3441000	412	708	297	0	47.30
		Effluent	0	292	25	4	2	11.3
WWTP2		Influent	3441000	412	708	297	0	47.3
		Effluent	9804	316	72	30	8	27.2
WWTP3		Influent	7170000	673	673	283	0	57.10
		Effluent	2755	498	16	2	1	36.2
WWTP4		Influent	2230000	572	139	99	40	37.1
		Effluent	141360	569	27	8	2	44.3
WWTP1	FEB	Influent	7800000	502	1126	519	95	33.40
		Effluent	6	829	33	10	2	3.1
WWTP2		Influent	9207	394	185	261	37	3.7
		Effluent	10760000	500	98	64	27	31.3
WWTP3		Influent	8130000	514	589	366	84	11.60
		Effluent	0	367	19	1	1	8.7
WWTP4		Influent	2850000	607	95	126	24	32.9
		Effluent	387300	559	34	13	2	34.9
WWTP1	MAR	Influent	8260000	557	1263	532	0	27.20
		Effluent	2	307	28	4	1	1.2
WWTP2		Influent	5120000	433	654	371	0	29.6
		Effluent	504	252	45	17	1	25.2

**WWTP3**		**Influent**	6690000	539	470	204	0	2.30
		**Effluent**	1	491	18	7	8	11.5
**WWTP4**		**Influent**	5380000	529	29	73	38	35.0
		**Effluent**	111990	464	108	8	2	36.9
**WWTP1**	**APR**	**Influent**	14210000	66	1597	473	247	3.00
		**Effluent**	2	48	32	4	2	27.2
**WWTP2**		**Influent**	18420000	58	430	231	158	42.6
		**Effluent**	7270	44	32	5	2	27.8
**WWTP3**		**Influent**	8164000	494	438	402	0	155.10
		**Effluent**	15	55	73	62	30	158.9
**WWTP4**		**Influent**	11370000	430	101	119	37	41.0
		**Effluent**	173290	306	26	9	2	37.8
**WWTP1**	**MAY**	**Influent**	17326000	478	415	237	0	45.80
		**Effluent**	6	388	27	4	1	4.8
**WWTP2**		**Influent**	9804000	508	345	90	75	40.6
		**Effluent**	1267	331	34	10	4	17.3
**WWTP3**		**Influent**	19863000	577	1190	393	0	6.40
		**Effluent**	9	519	59	47	18	33.0
**WWTP4**		**Influent**	6240000	511	192	164	63	40.3
		**Effluent**	198630	460	30	6	3	21.1
**WWTP1**	**JUNE**	**Influent**	19863000	338	234	178	67	25.50
		**Effluent**	0	337	24	3	3	6.4
**WWTP2**		**Influent**	5380000	479	671	582	0	30.0
		**Effluent**	11193	332	29	11	2	19.3
**WWTP3**		**Influent**	10120000	625	1250	352	206	17.80
		**Effluent**	47	497	25	5	3	36.5
**WWTP4**		**Influent**	8860000	401	110	266	0	44.1
		**Effluent**	189200	475	22	6	0	35.4
**WWTP1**	**JULY**	**Influent**	7701000	335	228	124	21	63.40
		**Effluent**	0	256	21	3	1	41.7
**WWTP2**		**Influent**	3873000	458	315	141	89	24.8
		**Effluent**	155310	370	33	4	3	30.5
**WWTP3**		**Influent**	9208000	488	476	312	0	14.20
		**Effluent**	308	305	28	20	14	10.6
**WWTP4**		**Influent**	41060000	410	433	291	71	60.2
		**Effluent**	15650	386	28	7	3	61.4
**WWTP1**	**AUG**	**Influent**	51720000	574	673	343	0	29.70
		**Effluent**	115	230	21	2	1	30.2
**WWTP2**		**Influent**	2950000	473	238	307	0	29.2
		**Effluent**	198630	705	32	10	2	29.8
**WWTP3**		**Influent**	86640000	524	631	288	0	0.10

		**Effluent**	0	398	12	2	1	30.3
**WWTP4**		**Influent**	155310000	445	623	300	143	37.8
		**Effluent**	173290	444	28	6	3	35.7
**WWTP1**	**SEPT**	**Influent**	12997000	638	1982	4560	0	31.90
		**Effluent**	27550	424	26	3	1	19.1
**WWTP2**		**Influent**	6488000	498	373	69	32	22.9
		**Effluent**	46110	251	26	3	1	29.7
**WWTP3**		**Influent**	26130000	593	1178	271	80	19.50
		**Effluent**	8	388	20	4	2	38.5
**WWTP4**		**Influent**	14136000	554	304	140	67	44.1
		**Effluent**	4950	541	28	7	3	41.9
**WWTP1**	**OCT**	**Influent**	19863000	525	1047	300	0	25.00
		**Effluent**	1	347	35	4	1	13.5
**WWTP2**		**Influent**	15531000	655	543	183	0	55.4
		**Effluent**	39900	460	28	4	2	23.3
**WWTP3**		**Influent**	2143000	689	344	263	97	22.90
		**Effluent**	0	559	28	21	3	
**WWTP4**		**Influent**	7701000	578	5786	141	58	46.6
		**Effluent**	920800	528	30	11	4	36.0

Figure S1. Enteric viruses Cq values comparison between PMAxx-RT-qPCR and ISC-RT-qPCR capsid integrity assays on influent and effluent wastewater samples from a single WWTP during a one-year period.

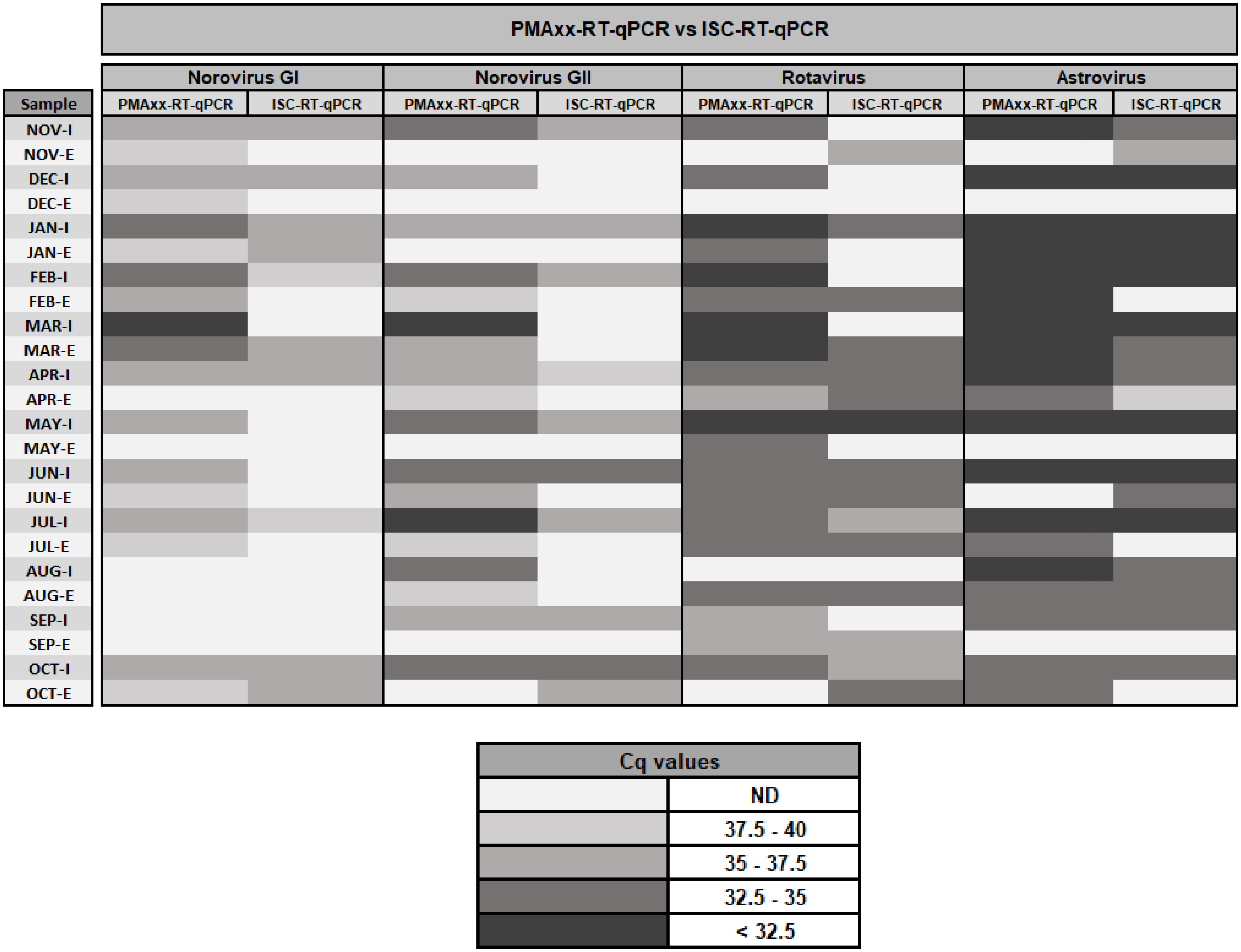

References

ISO - ISO 15216-1:2017 - Microbiology of the food chain — Horizontal method for determination of hepatitis A virus and norovirus using real-time RT-PCR — Part 1: Method for quantification [WWW Document], n.d. URL https://www.iso.org/standard/65681.html (accessed 6.2.21).

Jothikumar, N., Kang, G., Hill, V.R., 2009. Broadly reactive TaqMan® assay for real-time RT-PCR detection of rotavirus in clinical and environmental samples. J. Virol. Methods 155, 126–131. https://doi.org/10.1016/j.jviromet.2008.09.025

Stachler, E., Kelty, C., Sivaganesan, M., Li, X., Bibby, K., Shanks, O.C., 2017. Quantitative CrAssphage PCR Assays for Human Fecal Pollution Measurement. Environ. Sci. Technol. 51, 9146–9154. https://doi.org/10.1021/acs.est.7b02703
